# ANGPTL3 and residual atherosclerotic risk: from lipid metabolism to therapeutic targeting

**DOI:** 10.3389/fendo.2025.1706091

**Published:** 2026-01-05

**Authors:** Shuwei Weng, Chen Ding, Jinxiu Lin, Dajun Chai

**Affiliations:** 1 Cardiovascular Department, The First Affiliated Hospital, Fujian Medical University, Key Laboratory of Metabolic Heart Disease in Fujian Province, Clinical Research Centre of Metabolic Cardiovascular Disease in Fujian Province, Fuzhou, China; 2 Cardiovascular Department, National Regional Medical Center, Binhai Branch of the First Affiliated Hospital, Fujian Medical University, Fuzhou, China

**Keywords:** angiopoietin-like protein 3, lipid metabolism, triglyceride, cholesterol, atherosclerosis

## Abstract

Despite achieving recommended low-density lipoprotein cholesterol (LDL-C) targets, many patients remain at high risk of cardiovascular events due to elevated triglyceride-rich lipoproteins and remnants. Angiopoietin-like protein 3 (ANGPTL3) has emerged as a promising therapeutic target for addressing this residual risk. As a liver-secreted regulator of lipoprotein metabolism, ANGPTL3 influences triglycerides, LDL-C, and high-density lipoprotein cholesterol through inhibition of lipoprotein lipase and endothelial lipase. Human genetic studies and pharmacologic interventions consistently show that ANGPTL3 inhibition improves lipid profiles and lowers apolipoprotein B–containing lipoproteins, independent of LDL receptor function. This review integrates biological, genetic, and clinical evidence, and provides an overview of novel ANGPTL3-targeted therapies, offering new perspectives for cardiovascular prevention and lipid management.

## 1 Introduction

Cardiovascular disease (CVD) has become the leading cause of mortality worldwide, accounting for more than 30% of all annual deaths, with atherosclerotic cardiovascular disease (ASCVD) occupying a predominant position (1). Projections based on the 2019 Global Burden of Disease data indicate that global deaths from CVD are expected to increase from 20.5 million in 2025 to 35.6 million in 2050 (2). At present, dyslipidemia—especially elevated low-density lipoprotein cholesterol (LDL-C) and triglycerides (TG)—is recognized as a major risk factor for ASCVD (3). Among these, effective management of LDL-C has long been considered an essential strategy for both primary and secondary prevention of ASCVD, and current guidelines recommend statin-centered lipid-lowering regimens (4). However, numerous studies have shown that even when patients achieve guideline-recommended LDL-C targets, there remains a substantial residual risk of cardiovascular events. In particular, elevated TG and postprandial triglyceride-rich lipoproteins (TRLs) further drive atherogenesis (5). This residual risk suggests that controlling LDL-C alone cannot fully eliminate cardiovascular risk and that additional therapeutic targets and strategies must be explored.

In recent years, with a deepening understanding of lipid-metabolic mechanisms, angiopoietin-like protein 3 (ANGPTL3) has become a research hotspot due to its pivotal role in regulating lipid metabolism. First identified by Conklin et al. in 1999 (6), ANGPTL3 is expressed predominantly in the liver and secreted into the circulation, where it regulates plasma lipoprotein metabolism by inhibiting lipoprotein lipase (LPL) and endothelial lipase (EL) (7). ANGPTL3 not only markedly affects TG and LDL-C levels but is also closely involved in the metabolism of high-density lipoprotein cholesterol (HDL-C), thereby participating broadly in the maintenance of plasma lipid homeostasis (8).

Large-scale genetic studies have further confirmed the importance of ANGPTL3 in lipid metabolism and the development of atherosclerosis. Human genetic studies show that individuals with loss-of-function variants in ANGPTL3 have significantly lower TG and LDL-C levels and a reduced risk of ASCVD (9). Based on these findings, investigators have proposed that inhibiting ANGPTL3 may be a novel approach to reduce cardiovascular risk. Multiple drug development programs targeting ANGPTL3 are underway, including monoclonal antibodies (10) and antisense oligonucleotides (11), both of which have shown favorable lipid-lowering effects in preclinical and clinical studies (12). Notably, the ANGPTL3 monoclonal antibody evinacumab has been approved by the U.S. Food and Drug Administration (FDA) and the European Medicines Agency (EMA) for the treatment of homozygous familial hypercholesterolemia (HoFH), further underscoring the considerable clinical potential of ANGPTL3 as a therapeutic target (13).

Although substantial progress has been made in elucidating the role of ANGPTL3 in lipid regulation, its precise mechanisms of action and the effects and safety of long-term ANGPTL3 inhibition on ASCVD risk require further investigation. Therefore, by reviewing recent basic and clinical advances related to ANGPTL3, this article comprehensively describes the physiological role, mechanisms of action, and potential clinical value of ANGPTL3 in lipid metabolism, with the aim of providing scientific evidence and research ideas for drug development targeting ANGPTL3 and its application in the prevention and treatment of cardiovascular disease.

## 2 Structure, physiological functions, and tissue expression of ANGPTL3

### 2.1 Overview of the ANGPTL family

The angiopoietin-like (ANGPTL) family consists of eight members, ANGPTL1 through ANGPTL8. They are secreted, glycosylated proteins with significant structural homology to angiopoietins and exhibit diverse physiological and pathological functions, contributing to inflammation, angiogenesis, cell death, senescence, and hematopoiesis, as well as tissue repair, maintenance, and homeostasis (14).

ANGPTL 1–7 share a common domain architecture—an N-terminal signal peptide, an N-terminal coiled-coil domain (CCD), and a C-terminal fibrinogen-like domain (FLD). By contrast, ANGPTL8 lacks the FLD and canonical disulfide/glycosylation motifs but is homologous to the N-terminal regions of ANGPTL3/4 (15, 16). Several reviews annotate an SE1-like LPL-binding segment in ANGPTL8 by sequence homology and in-silico analyses (17); however, direct functional epitope mapping of SE1 was originally established for ANGPTL3/4 (18). In line with this architecture, ANGPTL8 primarily potentiates ANGPTL3-mediated LPL inhibition via complex formation (19). Each ANGPTL domain mediates specific functions: the N-terminal signal peptide directs sorting and secretion; the CCD mediates protein–protein interactions and the reduction of LPL activity (20); full-length ANGPTLs containing this region, as well as their cleaved N-terminal fragments, can oligomerize (6). The C-terminal FLD is the most conserved among ANGPTLs and acts as a ligand-binding unit for putative receptors or matrices; its cleaved fragment is released as a monomer (21).

### 2.2 Structure and molecular features of ANGPTL3

ANGPTL3 is encoded by the human ANGPTL3 gene located on chromosome 1p31. The mature protein comprises 460 amino acids and is a secreted glycoprotein. Histological and database evidence consistently indicate that it is synthesized mainly in the liver and released into the circulation. As with other ANGPTLs, ANGPTL3 contains an N-terminal signal peptide, an N-terminal CCD, a linker peptide, and a C-terminal FLD. Comparative sequence and phylogenetic analyses indicate that the CCD and FLD are evolutionarily conserved across vertebrates (mammals, birds, teleosts). In particular, a highly conserved 12-amino-acid motif within the CCD of ANGPTL3/4 is required for LPL inhibition (22, 23). The CCD mediates interactions with substrates/targets and determines oligomerization, whereas the FLD provides a fibrinogen-like scaffold with potential receptor/matrix-binding sites. UniProt and the Human Protein Atlas concordantly annotate its length, secretory nature, and liver-enriched expression.

ANGPTL3 exists *in vivo* as both a full-length protein and proteolytic fragments generated by proprotein convertases at specific sites. In humans and mice, furin is the primary *in vivo* convertase for ANGPTL3, cleaving at a canonical site (RAPR^224↓TT) to produce the N-terminal LPL-inhibitory fragment (24). Classical studies show that recombinant and endogenous ANGPTL3 can be cleaved between Arg221 and Ala222 and between Arg224 and Thr225; the N-terminal fragments (residues 17–207 or 17–165) increase plasma TG *in vivo*, whereas the C-terminal FLD fragment does not. Thus, the N-terminal CCD fragment is considered the core responsible for LPL inhibition (25). Further domain mapping demonstrates that the SE1 segment within the N-terminus is critical for binding to and inhibiting LPL; blockade of this segment markedly weakens ANGPTL3’s ability to inhibit LPL and lowers plasma TG in mice. This conclusion has been validated for the homologous region of ANGPTL4, indicating conservation of N-terminal functional motifs between the two proteins (18).

Post-translational modifications finely tune ANGPTL3 cleavage and activity. *In vitro* and cellular studies have shown that polypeptide N-acetylgalactosaminyltransferase 2 (GALNT2) initiates O-glycosylation at Thr226, which blocks furin and other proprotein convertases from cleaving ANGPTL3, thereby altering the composition and functional state of circulating fragments. In mouse models, up- or down-regulation of Galnt2 reduces or enhances Angptl3 cleavage, respectively, indicating that this mechanism is not confined to *in vitro* systems (26). In addition, the ANGPTL3 CCD forms di-/oligomers, and oligomerization is thought to facilitate LPL inhibition; the N-terminus is the “necessary and sufficient” inhibitory unit, as consistently supported by structure–function studies and reviews (18, 27).

ANGPTL3’s physiological function also depends on its specific interaction with ANGPTL8. ANGPTL8 is synthesized and secreted predominantly by the liver; transcriptomic atlases classify human ANGPTL8 as liver-enriched/secreted, whereas in mice Angptl8 is expressed in both liver and adipose depots (17). Upregulated in the postprandial state, ANGPTL8 forms a complex with ANGPTL3 that markedly enhances the latter’s inhibition of LPL. Multiple studies show that either protein alone has limited inhibitory effect on LPL, whereas co-expression or complex formation achieves physiologically relevant inhibition. ANGPTL8 is, to some extent, functionally dependent on the presence of ANGPTL3 to exert inhibitory activity, suggesting that the ANGPTL3/8 complex is a key module governing the LPL in peripheral tissues (28, 29).

In sum, domain architecture, specific proprotein convertase cleavage sites, GALNT2-mediated O-glycosylation at Thr226, and complex formation with ANGPTL8 jointly determine the molecular forms and activity of ANGPTL3 in the circulation. Among these, the N-terminal CCD/SE1 constitutes the functional core, whereas post-translational modifications and oligomeric state provide dynamic regulatory dials.

### 2.3 Tissue distribution and regulatory mechanisms of ANGPTL3 expression

In this section, we summarize the key transcriptional/hormonal and post-translational mechanisms that regulate ANGPTL3 expression and processing.

At the transcriptional level, liver X receptor (LXR) is a key upstream regulator of ANGPTL3. Reporter assays *in vitro* and animal experiments *in vivo* have demonstrated that LXR/retinoid X receptor (RXR) activation directly binds and activates the DR4 element in the ANGPTL3 promoter, significantly increasing hepatic Angptl3/ANGPTL3 transcription and secretion (30). By contrast, insulin exerts an overall negative regulatory effect on ANGPTL3. In humans, hyperinsulinemic clamp studies show a rapid reduction in plasma ANGPTL3 not attributable to altered peripheral clearance, consistent with the interpretation that insulin suppresses hepatic ANGPTL3 transcription/secretion; in the same study, insulin downregulated ANGPTL3 mRNA/protein in human hepatocyte lines, forming a coherent *in vivo*/*in vitro* evidence chain (31). Earlier cell and animal studies likewise showed reduced hepatocyte Angptl3 expression and secretion with insulin treatment, elevated hepatic and plasma ANGPTL3 in insulin deficiency, and reversal upon insulin replacement, further supporting an insulin-suppression/insulin-deficiency-elevation relationship (32). A plausible mechanism is that LXR directly activates the ANGPTL3 promoter, whereas peroxisome proliferator-activated receptor-β/δ (PPARβ/δ) suppresses promoter activity at the same DR4 site; given that insulin signaling often remodels hepatic transcriptional networks via the phosphatidylinositol 3-kinase (PI3K)–AKT axis, antagonism between LXR and PPAR at the ANGPTL3 promoter provides a reasonable transcriptional intersection for insulin-mediated downregulation (33).

Leptin also downregulates ANGPTL3. Classical *in vivo* and *in vitro* evidence indicates that leptin deficiency or resistance is associated with elevated hepatic Angptl3 transcription and plasma ANGPTL3, whereas exogenous leptin reduces hepatocyte Angptl3 expression and plasma ANGPTL3. Direct leptin treatment of primary/immortalized hepatocytes suppresses Angptl3 mRNA and secretion, suggesting a direct inhibitory effect on hepatocyte-derived ANGPTL3 (32). In humans, patients with generalized lipodystrophy receiving metreleptin replacement therapy exhibited a rapid decline in plasma ANGPTL3, consistent in direction with animal and cellular data and supporting negative regulation of hepatocyte ANGPTL3 by the energy/adipose-reserve–leptin axis (34).

**Figure 1:**
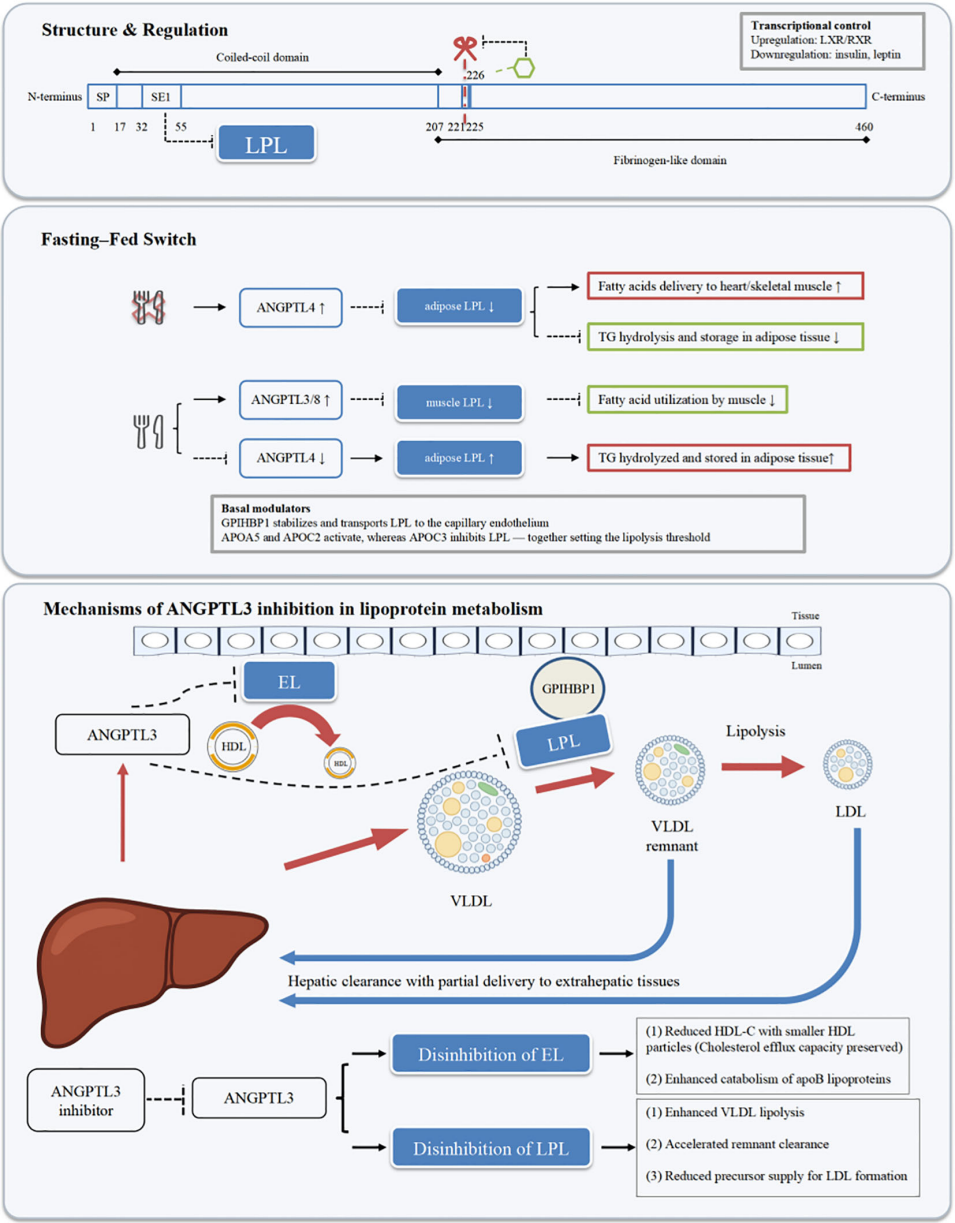
Overview of ANGPTL3 structure and its role in lipoprotein metabolism. The red scissor marks the furin cleavage site; the green hexagon indicates the O-glycosylation site at Thr226; SP denotes the signal peptide; SE1 represents the exon 1–encoded region in the N-terminal coiled-coil domain that mediates LPL binding.

At the level of protein secretion/processing, the ratio of full-length to cleaved ANGPTL3 is governed by the GALNT2-mediated Thr226 O-glycosylation/proprotein convertase cleavage axis. In primary hepatocytes and *in vivo* models, upregulating GALNT2 or inhibiting convertases markedly suppresses Angptl3 cleavage, whereas reducing Galnt2 expression promotes cleavage, underscoring this axis as a core mechanism determining the molecular-form spectrum of circulating ANGPTL3 (35). Notably, the physiological potency of ANGPTL3 also depends on its co-expression and complex formation with ANGPTL8, which is more readily upregulated under fed/insulin-replete states—thereby altering ANGPTL3’s effective activity and tissue-specific gating. Although this belongs to functional extension, in the context of expression control it emphasizes that tissues, hormones, and post-translational modifications jointly shape ANGPTL3’s biological effects. The structure and regulatory motifs of ANGPTL3 are illustrated in **Figure 1**, providing a visual bridge to the mechanistic pathways described in the following section.

## 3 Mechanisms of ANGPTL3 in lipid metabolism

### 3.1 Regulation of triglyceride metabolism

Intravascular clearance of TG mainly occurs on the luminal surface of capillaries, where LPL hydrolyzes chylomicrons (CM) and very-low-density lipoproteins (VLDL) (36). LPL activity is not constant but is dynamically regulated by endogenous inhibitors and activators (19). Among these, the ANGPTL3/4/8 axis is particularly influential: ANGPTL3 is a liver-derived inhibitor; ANGPTL8 is upregulated in the postprandial state and acts synergistically with ANGPTL3; ANGPTL4 is broadly expressed across metabolically active tissues (adipose, liver, skeletal muscle, heart, intestine) and operates in a nutrition- and tissue-dependent manner (28). During fasting in humans, adipose ANGPTL4 increases while adipose LPL activity declines, and with exercise ANGPTL4 can be induced (liver/muscle context), together supporting a dynamic, context-dependent inactivation of LPL. By altering effective LPL activity across tissues, this axis allocates lipid fluxes between postprandial and fasting states and is pivotal for determining plasma TG levels and tissue substrate supply. Genetic, pharmacological, and biochemical evidence in mice and humans consistently shows that ANGPTL3 inhibits LPL, slows VLDL-TG clearance, and raises plasma TG. This causal chain was first established by *in vivo* tracing and recombinant protein studies and has since been repeatedly validated and extended (37). Clinically, first-in-human and hypertriglyceridemia trials with the ANGPTL3 monoclonal antibody evinacumab demonstrated dose-dependent TG reductions with favorable safety (38).

Mechanistically, ANGPTL-mediated inhibition of LPL is not simply competitive site blocking; rather, it induces conformational inactivation, shifting LPL from an active to an irreversibly low-activity state. For ANGPTL4, hydrogen–deuterium exchange, cryo-EM, and functional data show that it catalyzes irreversible unfolding of the LPL α/β-hydrolase domain, causing loss of activity; when LPL is anchored to the endothelial protein glycosylphosphatidylinositol-anchored high-density lipoprotein–binding protein 1 (GPIHBP1), its structure is stabilized and it is markedly protected from ANGPTL4-mediated inactivation. GPIHBP1 ferries LPL from parenchymal cells to the capillary lumen and, through its acidic intrinsically disordered domain, stabilizes LPL, reduces its susceptibility to ANGPTL3/4, and allows LPL to migrate within the glycocalyx to expand contact with TG-rich lipoproteins. This dynamic inactivation–protection interplay constitutes the fundamental lipolytic architecture at the capillary interface (39, 40).

During fasting, the organism must prioritize fatty acid supply to oxidative tissues such as heart and skeletal muscle. ANGPTL4 is induced in adipose tissue to inactivate LPL locally, thereby reducing TG uptake into adipose depots and channeling substrates to energy-consuming tissues. Animal and transcriptional-kinetic studies repeatedly confirm this: ANGPTL4 rises rapidly early in fasting, with a concomitant fall in adipose LPL activity; in Angptl4-deficient mice this rapid response is markedly blunted or absent (41). Meanwhile, the hepatokine apolipoprotein A5 (APOA5) cooperates with endothelial glycosaminoglycans and LPL to maintain and bridge an effective lipolytic platform, and reduces ANGPTL3/8-mediated LPL inhibition—acting as a stabilizer of the lipolytic platform and an attenuator of ANGPTL3/8-mediated inhibition during the fasted–fed transition. In human and animal models, APOA5 deficiency lowers capillary LPL abundance/activity and yields a phenotype of relatively excessive ANGPTL3/8 activity (42, 43).

Upon feeding, ANGPTL8 is rapidly upregulated by insulin and nutrient signals and complexes with hepatic ANGPTL3. Numerous independent lines of evidence show that ANGPTL8 alone has limited inhibitory effect on LPL; significant inhibition and increased plasma TG occur only in the presence of ANGPTL3. The complex preferentially imposes a brake on oxidative tissues (skeletal/heart muscle), while ANGPTL4 is downregulated and LPL is opened in adipose tissue. The net effect is to divert postprandial TG toward adipose storage, avoiding competition with oxidative tissues for substrates (29). Pulse-label hydrogen–deuterium exchange mass spectrometry indicates that ANGPTL3/8, like ANGPTL4, induces ATP-independent conformational unfolding of LPL, offering a biophysical explanation for rapid, state-dependent shifts in LPL activity in the postprandial state (44).

Plasma apolipoproteins further modulate these processes. APOC2 is an essential LPL cofactor, whereas APOC3, in concert with the ANGPTL axis, raises the opening threshold to restrain excessive lipolysis (45). The endoplasmic reticulum (ER)-resident transcription factor cAMP-responsive element-binding protein H (CREBH) upregulates APOC2/APOA5 and other lipolysis-promoting genes, systemically lowering plasma TG and coupling regulation to circadian/nutritional status—thereby calibrating the transcriptional set-point of LPL activity (45). These time- and tissue-dependent adjustments, superimposed on the GPIHBP1–LPL–glycocalyx carrier/migration microenvironment, ultimately determine the destination and rate of TG flux and explain why substrates preferentially fuel oxidative tissues during fasting and are directed to adipose storage after feeding. In short, ANGPTL3/4/8 dynamically balance conformational inactivation, protection, and substrate delivery at the capillary lumen to produce rapid, state- and tissue-dependent modulation of LPL activity; fasting emphasizes ANGPTL4-driven reduction of adipose LPL activity, whereas feeding emphasizes stronger inhibition in oxidative tissues by the ANGPTL3/8 complex.

### 3.2 Effects on cholesterol metabolism

With respect to cholesterol metabolism, ANGPTL3 can be summarized as a dual hub acting upstream and downstream: on one end, it inhibits EL, thereby affecting HDL composition and turnover; on the other, it accelerates lipolysis and disposal of TG-rich lipoproteins, reducing the VLDL-to-LDL cascade and thus lowering LDL-C—an effect that is, to a considerable extent, independent of the LDL receptor (LDLR). Genetic and experimental data establish EL as a key determinant of HDL-C: overexpression of EL lowers HDL-C, whereas knockout or loss-of-function variants in LIPG raise HDL-C in mice and humans; EL can also participate in triglyceride-rich lipoprotein catabolism under nutrient-excess states (46–48). For HDL, early evidence in humans and animals clearly shows that ANGPTL3 directly inhibits the phospholipase activity of EL: Angptl3-deficient mice exhibit markedly reduced HDL-C and HDL phospholipids, whereas ANGPTL3 reconstitution restores these parameters, establishing the ANGPTL3–EL–HDL causal chain (49). *In vitro*, ANGPTL3’s inhibition of EL is dose- and temperature-dependent, and is attenuated when EL is anchored on endothelial surfaces or in the presence of heparin/heparan sulfate proteoglycans, suggesting that vascular microenvironment and enzyme anchoring modulate effect strength (50). Thus, genetic or pharmacologic inhibition of ANGPTL3 is expected to disinhibit EL and lower HDL-C—an observation repeatedly confirmed across modalities and populations. Importantly, decreased HDL-C in the context of ANGPTL3 inhibition/deficiency does not necessarily imply impaired cholesterol efflux capacity; some studies even note selective binding/function remodeling of ANGPTL3 with HDL particles, hinting at a partial decoupling of HDL concentration and function. Nonetheless, HDL proteomics and functional changes under different metabolic states require further evaluation (51).

Effects on LDL are of greater translational significance. Inhibiting ANGPTL3 lowers LDL-C even when LDLR function is impaired, as demonstrated by randomized and long-term studies: in HoFH, evinacumab significantly reduces LDL-C on top of standard therapy and concomitantly lowers non-HDL-C and total cholesterol, confirming an LDLR-independent clinical phenotype (10). Mechanistically, cross-species research suggests that the principal action of ANGPTL3 inhibition occurs upstream of LDL formation: by accelerating VLDL lipolysis and remnant clearance, shrinking VLDL particle size, and reducing lipid cargo, it reduces the substrate feeding into the LDL pathway. In mice, the LDL-C reduction induced by ANGPTL3 inhibition is markedly blunted or abolished when EL is absent, indicating that EL is a key mediator of LDLR-independent LDL lowering (52). Isotopic kinetic studies show that evinacumab increases the fractional catabolic rates of intermediate-density lipoprotein (IDL)-apoB and LDL-apoB; in some subjects it also lowers VLDL-apoB production rates—together contributing to LDL-C reduction (53).

Regarding non-HDL indices, changes upstream of LDL formation are reflected in non–high-density lipoprotein cholesterol (non-HDL-C), VLDL-C/IDL-C, and remnant cholesterol: in mixed hyperlipidemia or elevated-TG populations, antisense oligonucleotide (ASO) or small interfering RNA (siRNA) inhibition of ANGPTL3 significantly reduces VLDL-C and remnant cholesterol in a dose-responsive manner (54). This phenotype indicates that inhibiting ANGPTL3 promotes faster hydrolysis of TRLs and their conversion into remnants amenable to hepatic uptake, thereby lowering total apolipoprotein B (apoB) burden and non-HDL-C and reducing exposure to apoB-containing lipoproteins overall (55). As for lipoprotein(a) [Lp(a)], ANGPTL3’s effects are limited and heterogeneous: in high-risk populations with impaired LDLR function, evinacumab may modestly reduce Lp(a), but the magnitude is small and inter-individual variability is large—insufficient to justify it as a primary treatment target or criterion (10).

### 3.3 Direct effects of ANGPTL3 on hepatic lipid metabolism

Although ANGPTL3’s classic action site is the vascular lumen—modulating TRL mobilization and partitioning by inhibiting LPL/EL at peripheral tissues—growing evidence indicates that ANGPTL3 inhibition or deficiency exerts independent metabolic effects on the hepatic side of the liver–vasculature axis. On one hand, it alters the lipid load of VLDL assembled and secreted by hepatocytes; on the other, it remodels interactions between TRLs/remnants and the liver, accelerating their disposal in ways not reliant on a single receptor and thereby, as feedback, reducing LDL formation (53). This kinetic signature aligns with the clinical phenotype of LDLR-independent benefit observed with evinacumab in HoFH (10).

At the hepatic output level, an ANGPTL3 monoclonal antibody reduces VLDL-TG secretion by 61% in mice without changing VLDL apoB-100 particle number; in other words, particle number is unchanged but TG cargo per particle is reduced. The same study reported no changes in intrahepatic TG content, *de novo* lipogenesis (DNL), or fatty acid oxidation (FAO), suggesting that lower VLDL-TG may not arise from endogenous suppression of synthesis/oxidation but rather reflects reduced availability of lipid substrates for assembly—such as diminished peripheral FFA return or remnant lipids—or a systemic effect whereby particles are cleared more rapidly and do not accumulate in the circulation to become LDL. The authors posited that ANGPTL3 inactivation does not change particle number but alters composition and accelerates clearance, thereby reducing the substrate entering and the residence time within the LDL-generating pathway and consequently suppressing LDL formation (56).

Proceeding downstream to hepatic uptake and clearance, ANGPTL3 inhibition produces remnant particles with smaller size, lower surface phospholipid/TG load, and remodeled apolipoproteins, which enhances their adhesion to and endocytosis by hepatic clearance machinery. Prior work on hepatic receptors has shown that the heparan sulfate proteoglycan (HSPG) system—especially syndecan-1 (SDC1)—is central to hepatocyte clearance of chylomicron/VLDL remnants. Multiple mouse and *in vitro* studies confirm that HSPG/SDC1 can bind and internalize remnant particles bridged by LPL, constituting an important LDLR-independent clearance route (57–59). Notably, mechanistic studies targeting ANGPTL3 also suggest redundancy in clearance pathways: in mice with liver-specific deletions of Ldlr, Lrp1, or Sdc1, neutralizing endogenous ANGPTL3 still significantly reduces plasma cholesterol and TG and accelerates non-canonical clearance. This implies that remnant ingestibility is broadly enhanced after ANGPTL3 inhibition—not by upregulating a single receptor pathway but by altering particle physicochemical properties and surface protein assembly to improve the performance of multiple clearance routes simultaneously (56).

From the perspective of hepatic cholesterol reuptake and reverse cholesterol transport (RCT), ANGPTL3 blockade not only reduces atherogenic lipids but also increases hepatic uptake/clearance of HDL-labeled cholesterol and enhances overall RCT flux. Although HDL-C levels usually decline, RCT-related function is not impaired and is even augmented in murine macrophage-to-feces tracer models. This has been directly verified in ASO studies of ANGPTL3: HDL-labeled lipids are cleared more rapidly and taken up more by the liver in ASO-treated mice, indicating that ANGPTL3 inhibition enhances hepatic handling of cholesterol and confers benefits at the hepatic end of the RCT pathway (60).

Finally, regarding long-term hepatic lipid safety of ANGPTL3 inhibition, genetic and imaging data indicate that individuals with homozygous or heterozygous ANGPTL3 deficiency display broad hypolipidemia but do not have increased hepatic fat content by quantitative MRI compared with wild-type individuals. This observation provides epidemiologic and imaging-based safety signals for long-term hepatic targeting of ANGPTL3 (61).

## 4 Genetic variation of ANGPTL3, population lipid levels, and cardiovascular risk

### 4.1 ANGPTL3 mutations and changes in lipid levels

Evidence from human genetics indicates that loss-of-function (LoF) variants in ANGPTL3 produce a combined hypolipidemic phenotype characterized by concomitant reductions in multiple lipoproteins, with a clear dose–response pattern. Early exome-sequencing work identified homozygous, heterozygous, and nonsense variants of ANGPTL3 within a single pedigree; individuals with homozygous or heterozygous variants had significantly lower plasma LDL-C, HDL-C, and TG, with smaller reductions in heterozygotes. Stratified analysis within the same pedigree visually demonstrated the correspondence between different alleles and stepwise decreases in lipid parameters, providing initial human genetic evidence for the causal link between ANGPTL3 deficiency and combined hypolipidemia (62). In large cohorts using exome sequencing–phenotype association, this model was quantitatively confirmed. In the DiscovEHR cohort, 13 predicted LoF variants (four nonsense, six frameshift indels, and three splice-site variants) were aggregated; heterozygous carriers had a median 27% lower TG, 9% lower LDL-C, and 4% lower HDL-C, and circulating ANGPTL3 protein itself was about half that of controls. The carrier frequency for these LoF variants was approximately 1 in 237, indicating that such variants are not exceedingly rare (9).

Studies from the Italian Campodimele population and multiple pedigrees further clarified the relationship between “autosomal recessive complete deficiency leading to pan-hypolipidemia” and “heterozygous partial deficiency causing a milder phenotype.” Truncating variants represented by S17X rendered plasma ANGPTL3 nearly undetectable in homozygotes/compound heterozygotes and were accompanied by broad, marked reductions in LDL-C, TG, and HDL-C (63). In heterozygotes, ANGPTL3 protein decreased only partially, with milder lipid changes—often decreased total cholesterol and HDL-C—with the magnitude of LDL-C and TG reduction depending on variant type (64). These data indicate that complete LoF of ANGPTL3 leads to autosomal recessive combined hypolipidemia, with most pathogenic variants being N-terminal truncations or key splice defects, although effects on LDL-C, TG, and HDL-C vary by position/type.

Beyond standard lipid measures, ANGPTL3 deficiency exerts a “threshold-like” effect on lipoprotein particle number and subclasses. Systematic analysis across 19 families showed that significant correlations between lipoprotein particle concentrations (VLDL-p, LDL-p, HDL-p) and total cholesterol, TG, HDL-C, and ANGPTL3 levels emerged only when plasma ANGPTL3 fell to less than one quarter of normal (a cut point of 60 ng/mL); VLDL-p displayed a higher sensitivity threshold of 58%. This suggests that during modest reductions in plasma ANGPTL3, lipid changes are mild; only with large decreases or deficiency are the full combined effects on peripheral lipolysis and transport fully expressed, yielding broad hypolipidemia (65). This aligns with ANGPTL3’s physiological role in governing TRL generation/clearance and HDL turnover via peripheral LPL/EL regulation. The above genetic–phenotypic landscape provides strong human evidence and baseline expectations for interpreting cholesterol stratification, remnant metabolism, and lipid changes upon pharmacologic inhibition of ANGPTL3.

### 4.2 Associations between ANGPTL3 variants and cardiovascular disease risk

Current human genetic evidence overall supports the causal chain whereby lifelong reduction in ANGPTL3 function lowers multiple lipoproteins (especially TG and apolipoprotein B–related particles) and, in turn, reduces ASCVD risk. In an early multi-cohort exome-sequencing study centered on DiscovEHR, among 13,102 patients with coronary artery disease (CAD) and 40,430 controls, carriers of ANGPTL3 LoF variants had an odds ratio (OR) for CAD of 0.59, replicated across four independent populations; the same paper used pharmacologic antagonism in humans and atherosclerotic mice for causal validation, forming an evidence chain from genetics to pharmacology to atherosclerosis phenotypes (9).

In broader populations, three lines of evidence—pedigrees, heterozygous deficiency in the general population, and plasma-level stratification in myocardial infarction case–control studies—converge on a protective effect: heterozygous ANGPTL3 LoF carriers had 34% lower CAD risk. In case–control analyses without genetic typing, individuals in the lowest tertile of circulating ANGPTL3 also exhibited significantly lower myocardial infarction risk (adjusted OR 0.65). These findings are consistent with concurrent reductions in TG, LDL-C, and HDL-C in LoF carriers, suggesting an epidemiologically coherent link between ANGPTL3’s multi-lipoprotein effects and reduced ASCVD risk (66).

More recent target-oriented Mendelian randomization (MR) analyses introduce nuance. When protein-quantitative or hepatic expression variants are used as instruments, reductions in ANGPTL3 associated with common variants do not show a clear association with reduced CAD risk. However, when protein-truncating variants and other strong LoF instruments are meta-analyzed, the per-allele CAD risk ratio is 0.71, closer in direction and magnitude to earlier sequencing studies. This instrument-dependence likely reflects an effect-size threshold/dose–response: common variants tend to produce modest downregulation of ANGPTL3, diluting downstream effects on apoB and CAD; by contrast, LoF variants cause lifelong, larger reductions that more closely mirror the biology under strong pharmacologic inhibition (67). Another MR study observed that with protein-quantitative or hepatic expression instruments, ANGPTL3 downregulation robustly lowers TG and has weaker impact on apoB, with no consistent associations with CAD or ischemic stroke. In PTV analyses based on UK Biobank, event rates for carriers versus noncarriers were similar (10.2% vs. 10.9%), suggesting that when apoB reduction is insufficient, net effects on clinical endpoints may be small (68). A reasonable inference is that clinical benefit may require near-complete inhibition of ANGPTL3—echoing the aforementioned threshold phenomenon.

It is important to note that cohort studies based on plasma ANGPTL3 levels do not always align with genetic findings. In patients with CAD or acute coronary syndrome (ACS), higher fasting ANGPTL3 levels have been associated with increased risk of recurrent events (69, 70). Yet when free ANGPTL3 and the ANGPTL3/8 complex are assayed separately—as shown in Swedish and other cohorts—the atherogenic lipoprotein profile and coronary heart disease (CHD) risk correlate with the ANGPTL3/8 complex rather than free ANGPTL3 (71). In the Ludwigshafen Risk and Cardiovascular Health study and the German epidemiological trial on ankle–brachial index (ABI), ANGPTL3/8 was not associated with cardiovascular mortality; in contrast, free ANGPTL3 was positively associated with cardiovascular mortality in ABI, and the C-terminal fragment of ANGPTL4 correlated with inflammation, diabetes prevalence, and cardiovascular mortality (72). These differences indicate that whether one measures free ANGPTL3, the ANGPTL3/8 complex, or other specific fragments substantially affects the direction and magnitude of risk associations; concentrations are also sensitive to fed/fasted state, inflammation, and assay methodology. Therefore, when judging ANGPTL3’s suitability as a drug target, priority should be given to evidence from populations with lifelong functional deficiency and rigorous target-oriented MR, rather than inferring causality from one-time plasma levels.

Overall, current human genetics supports the proposition that inhibiting ANGPTL3 may reduce ASCVD risk, but the effect size depends on the degree of inhibition and its transmission to the apoB/remnant pathway. Strong, near-complete functional inhibition accompanies more pronounced reductions in apoB and remnant cholesterol, yielding sizable decreases in CAD risk; mild “weak inhibition” corresponding to common variants may mainly lower TG, with less evident benefits on clinical endpoints. High-level randomized trials directly testing event reduction are still lacking; aligning the causal direction inferred from LoF with the magnitude of pharmacologic lipid lowering is key to assessing the potential of ANGPTL3 inhibition to translate into outcome benefits.

### 4.3 Gene–environment interactions related to ANGPTL3

Gene–environment interactions involving ANGPTL3 have been observed in dietary composition, physical activity, and pharmacologic interventions, which influence ANGPTL3 levels, lipids, and ASCVD risk. Diet–gene evidence comes from both prospective and intervention studies. In the Korean Genome and Epidemiology Study (KoGES), a 10-year follow-up of rs11207997 showed that the T allele was associated with a reduced risk of diabetes, with significant interactions under specific food or nutrient intakes. When baseline TG was included in the model, the genetic effect was partially attenuated, suggesting that long-term TG load may be a key bridge linking ANGPTL3 variation to glycemic risk, as supported by Cox regression models using population data (73). In a randomized, controlled feeding study, rs10889337 interacted with dietary polyunsaturated fatty acids (PUFA): under a high-PUFA diet, different genotypes exhibited divergent reductions in total cholesterol and apoB (interaction P ≈ 0.01 and 0.006, respectively), implying that certain genotypes may derive greater lipid benefits (74). Similarly, short-term dietary composition can differentially modulate the ANGPTL3 axis by sex: in a 7-day randomized trial comparing PUFA- versus saturated fatty acid (SFA)-rich diets, women on an SFA diet had smaller changes in ANGPTL3/ANGPTL8 and lower postprandial TG peaks, whereas men did not show the same pattern—suggesting that biological sex may be an important environmental modifier of ANGPTL3-related phenotypes and motivating future three-way interaction analyses among gene, sex, and diet (75). Beyond fatty acid intake, high-fructose diets in humans and nonhuman primates increased circulating ANGPTL3 by 30–40% and remodeled TG-rich lipoprotein metabolism, providing time-series evidence that high sugar loads can amplify the TG-raising effects of the ANGPTL3 axis. In acute oral glucose tolerance test (OGTT) and oral lipid tolerance test (OLTT) challenges, ANGPTL3 fluctuated over hours in a reproducible manner, indicating rapid and reversible responses to nutrient stimuli. Thus, dietary composition can significantly modulate activation of the ANGPTL3 pathway, producing different phenotypes for the same genotype under different dietary patterns (76).

As for physical activity, available evidence indicates that exercise—both in short postprandial windows and over the longer term—can attenuate ANGPTL3-axis inhibition of LPL. Medium- to long-term aerobic or combined training lowers circulating ANGPTL3/ANGPTL8, increases LPL activity, reduces plasma TG and apoB, and decreases visceral fat and fasting insulin (77). A single bout of moderate postprandial exercise reduces hepatic ANGPTL8, lowers ANGPTL3 recruitment to LPL sites on myocardial/skeletal muscle endothelium, and thereby relieves LPL inhibition, enhancing muscular utilization of TG-derived fatty acids (78). Although large-scale interaction statistics between ANGPTL3 loci and exercise are lacking, these interventions imply that in LoF carriers or low-expression phenotypes, exercise may further amplify beneficial reductions in TG or apoB; conversely, in high-expression or high-risk phenotypes, exercise can serve as an environmental “hedge,” lowering effective ANGPTL3 activity.

In addition to diet and physical activity, pharmacologic interventions are important modulators of the ANGPTL3 axis. Real-world and small trial data suggest that statins reduce plasma ANGPTL3 by 15–20% in hypercholesterolemic patients—possibly related to dampened hepatic LXR signaling (79). At the target level, ANGPTL3 inhibitors and statins or PCSK9 inhibitors display additive apoB-lowering effects (80). Thus, under a genetic background of partial ANGPTL3 deficiency or low expression, conventional lipid-lowering drugs may further depress ANGPTL3-axis activity and modify the phenotypic expression of genetic effects; conversely, in high-expression individuals, pharmacotherapy may partially offset unfavorable genetic backgrounds. Validation of these inferences will require genotype-stratified intervention studies.

In summary, existing evidence clearly indicates that external interventions influence the ANGPTL3 axis: when diet, activity, and drugs push the system toward lower effective ANGPTL3 activity, the same genotype yields amplified benefits along TG and apoB pathways; under unfavorable environments such as high sugar or high SFA intake, upregulation of the ANGPTL3 axis may dilute genetic protection or obscure it with phenotypic noise. Looking ahead, integrating LoF and event-risk causality with stratification by diet/exercise/pharmacotherapy may advance ANGPTL3 from a “druggable target” to a “precision cardiometabolic target amenable to stratified management”.

## 5 Advances in pharmacologic inhibition of ANGPTL3

### 5.1 Monoclonal antibodies

Evinacumab, approved in 2021 by the FDA and EMA, is a humanized immunoglobulin G4 monoclonal antibody. By binding ANGPTL3, evinacumab increases LPL and EL activities, resulting in reductions in plasma TG, LDL-C, and HDL-C. Although the precise mechanism for LDL-C lowering remains incompletely defined, both animal (52) and clinical (10) data indicate that the effect is independent of the LDLR; in mice lacking both LDLR and EL, inhibition of ANGPTL3 does not effectively lower LDL-C, suggesting that EL is required for LDLR-independent LDL-C reduction (81).

In a phase I randomized, double-blind, placebo-controlled study in healthy Japanese and White volunteers (n=96; baseline LDL-C 100–160 mg/dL), participants received subcutaneous (SC) evinacumab 300 mg as a single dose or once weekly for eight weeks, or intravenous (IV) evinacumab 5 mg/kg or 15 mg/kg every four weeks for two doses (82). Evinacumab was well tolerated across all regimens, with no serious adverse events (SAEs). Common adverse events were injection-site reactions, headache, and nasopharyngitis (predominantly mild to moderate) at rates similar to placebo, and pharmacokinetics were comparable between Japanese and White participants. Lipid effects were dose-dependent. In the 15 mg/kg IV cohort, by day 57, TG decreased by as much as 63.1%, LDL-C by 33.6%, non–HDL-C by 34.2%, apolipoprotein B (apoB) by 27.0%, and total cholesterol (TC) by 29.4%. In the 5 mg/kg IV cohort, corresponding decreases were 32.5% (TG), 18.2% (LDL-C), 20.4% (non–HDL-C), 18.7% (apoB), and 19.4% (TC). SC dosing produced similar patterns; for example, 300 mg weekly for eight weeks reduced LDL-C by 26.2% and TG by 36.9%. Reductions in TG were evident by day 2 after dosing and reductions in LDL-C by day 3, with both effects sustained to day 57.

Two additional phase I studies in patients with hypertriglyceridemia further corroborated the lipid-lowering profile and safety of evinacumab (83). In the single-ascending-dose study (NCT01749878; n=83; baseline TG 150–450 mg/dL, LDL-C ≥100 mg/dL), a single SC or IV administration was followed for 126 days: in the IV 250 mg group at day 15, TG decreased by 47.5%, LDL-C by 22.3%, non–HDL-C by 24.6%, apoB by 22.0%, TC by 19.1%, and apolipoprotein C-III (apoC-III) by up to 67.5%; HDL-C decreased by approximately 13.6%. In the multiple-ascending-dose study (NCT02107872; n=56; follow-up to 183 days), the maximal observed decreases included TG by 64.8% with SC 300 mg weekly and by 69.9% with IV 15 mg/kg every four weeks; additional reductions included LDL-C by 27.9%, non–HDL-C by 35.3%, apoB by 33.5%, TC by 30.9%, and apoC-III by 75.3%. Across doses and routes, safety remained favorable, with no treatment-related SAEs and mostly mild/moderate events, and no signal of dose-accumulation toxicity.

### 5.2 Antisense oligonucleotides and small interfering RNA

Vupanorsen is a second-generation N-acetylgalactosamine (GalNAc)3-conjugated ASO targeting ANGPTL3 mRNA in the liver, thereby lowering circulating ANGPTL3 and improving lipid parameters. It has been evaluated in healthy volunteers, in patients with hypertriglyceridemia, in those with type 2 diabetes and metabolic disturbances, and in individuals with familial partial lipodystrophy.

Phase I pharmacokinetic/pharmacodynamic studies in Asia demonstrated consistent pharmacology and tolerability. In an open-label Chinese study (n=18), single SC doses of 80 mg or 160 mg reduced ANGPTL3 by 59.7% and 69.5%, respectively; TG by 41.9% and 52.5%; and non–HDL-C by 23.2% and 25.4%. Adverse events were mild, predominantly injection-site reactions, and no SAEs were reported (84). In a Japanese randomized, double-blind, placebo-controlled study, single doses of 80 mg or 160 mg reduced ANGPTL3 by 62.7–69.2%, TG by 48.1–50.4%, non–HDL-C by 25.3–41.8%, and LDL-C by 12.7–29.1%; tolerability was generally good (85).

In a phase IIa randomized, double-blind trial (n=105) in patients with type 2 diabetes, nonalcoholic fatty liver disease, and moderate hypertriglyceridemia, 24 weeks of vupanorsen (20 mg once weekly or 40/80 mg every four weeks) was associated with decreases in TG by 36–53%, ANGPTL3 by 41–59%, apoC-III by 58%, remnant cholesterol by 38%, non–HDL-C by 18%, and apoB by 9%. Injection-site reactions were the most common adverse events; no SAEs occurred, and liver enzyme elevations were generally mild (11). In an exploratory study in familial partial lipodystrophy (n=4; 20 mg weekly for 26 weeks), treatment was associated with decreases in TG by 59.9%, ANGPTL3 by 54.7%, VLDL-C by 53.5%, non–HDL-C by 20.9%, and free fatty acids by 41.7%, alongside improvements in adipose insulin resistance and postprandial lipid responses; no serious adverse effects or thrombocytopenia were observed (86).

The phase IIb TRANSLATE-TIMI 70 trial (n=286) enrolled high-risk atherosclerotic patients receiving background statins with non–HDL-C ≥100 mg/dL and TG 150–500 mg/dL. Over 24 weeks, vupanorsen administered at 60–160 mg every two weeks or 80–160 mg every four weeks produced a maximal decrease in ANGPTL3 of 95.2%. Across dose groups, TG decreased by 41.3–56.8%, non–HDL-C by 22.0–27.7%, LDL-C by 7.9–16.0%, and apoB by 6.0–15.1%. Components of the TG-rich lipoprotein fraction also declined substantially, with remnant cholesterol decreasing by 42–59% and VLDL-C by 52–67% in a dose-dependent manner that closely tracked the extent of ANGPTL3 suppression (54, 55). In a prespecified subgroup analysis, vupanorsen was associated with increases in hepatic fat fraction (HFF); at the highest dose, the relative increase reached 76%, corresponding to an absolute increase of 7.0 percentage points. Changes in HFF correlated moderately with elevations in ALT and aspartate aminotransferase (AST), indicating a dose-related risk of hepatic steatosis (87). Most adverse events were mild injection-site reactions and transaminase elevations; a small number of participants experienced ALT values exceeding three times the upper limit of normal, without clinical liver injury.

Population pharmacokinetic (PK) and PK–pharmacodynamic (PD) modeling based on phase I/II data indicated rapid absorption after SC administration, followed by distribution consistent with a two-compartment model and a terminal half-life of approximately 3–5 weeks. Model-based simulations suggested that a monthly dose of 320 mg would be expected to reduce ANGPTL3 by about 75%, although TG and non–HDL-C might not reach prespecified targets simultaneously (88).

Whether the observed increases in hepatic fat with vupanorsen reflect an on-target consequence of ANGPTL3 inhibition remains debated. Analyses of ANGPTL3-deficient carriers in the Campodimele and St. Louis cohorts and Mendelian randomization using UK Biobank indicated no excess risk of hepatic fat accumulation among carriers relative to noncarriers, suggesting that the HFF signal with vupanorsen may relate to molecule-specific chemistry or delivery rather than the target itself (61).

In parallel, small interfering RNA (siRNA) therapies have advanced into clinical testing. Zodasiran, a N-acetylgalactosamine (GalNAc)-conjugated siRNA, promotes degradation of ANGPTL3 mRNA via the RNA-induced silencing complex in hepatocytes. Compared with ASO therapy, siRNA approaches may afford longer duration and less frequent dosing. In a phase I basket study, single- and repeat-dose cohorts in healthy adults and individuals with hepatic steatosis showed that, in healthy participants, by day 85 after a single injection ANGPTL3 decreased by 45–78%, TG by 34–54%, and non–HDL-C by 18–29%. After two injections (100–300 mg, four weeks apart), by day 113 ANGPTL3 decreased by 64–93%, TG by 62–72%, non–HDL-C by 41–49%, LDL-C by 34–45%, and apoB by 28–39%. In participants with hepatic steatosis, repeat dosing at the same time point was associated with decreases in ANGPTL3 by 85%, TG by 44%, non–HDL-C by 37%, LDL-C by 35%, and apoB by 20%. No SAEs were observed; typical adverse events were mild injection-site reactions, headache, and transient ALT elevations. Magnetic resonance imaging (MRI) did not reveal increases in hepatic fat; some participants exhibited decreases (89).

The phase IIb ARCHES-2 trial (n=204) in mixed hyperlipidemia administered SC zodasiran 50 mg, 100 mg, or 200 mg at weeks 0 and 12 with follow-up to week 36. At week 24, relative to baseline and versus placebo, ANGPTL3 decreased by 54%, 70%, and 74% across the three dose groups. Corresponding decreases were 51%, 57%, and 63% for TG; 29%, 29%, and 36% for non–HDL-C; 16%, 14%, and 20% for LDL-C; and 19%, 15%, and 22% for apoB. Zodasiran was generally well tolerated, with mild injection-site erythema and headache being the most common events. A transient increase in glycated hemoglobin was observed in some individuals with diabetes at the 200 mg dose, warranting attention to glycemic safety in metabolically compromised populations (90).

Solbinsiran is a hepatocyte-targeted, GalNAc–conjugated siRNA designed to silence ANGPTL3 mRNA in the liver. In preclinical and first-in-human studies, single- and repeat-dose cohorts demonstrated dose-dependent reductions in circulating ANGPTL3 and atherogenic lipoproteins. In adults with mixed dyslipidemia, solbinsiran reduced ANGPTL3 by 76–86%, triglycerides by 61–73%, non–HDL-C by 33–41%, LDL-C by 21–30%, and apoB by 24–30%; the effects were durable after single or two doses, and the overall safety profile was acceptable, with most adverse events being mild injection-site reactions or laboratory abnormalities (91). In the double-blind, randomized, placebo-controlled PROLONG-ANG3 Phase 2 trial, adults with mixed dyslipidemia received subcutaneous solbinsiran (100–800 mg on day 1 with or without repeat dosing on day 90). At day 180, solbinsiran significantly reduced apoB by 23–30% compared with placebo (primary endpoint), alongside decreases in ANGPTL3 by 77–85%, TG by 59–70%, non–HDL-C by 28–37%, LDL-C by 17–25%, and VLDL-C by 46–54%. Importantly, hepatic fat fraction declined in the active-treatment groups, in contrast to the hepatic fat increases observed with vupanorsen. Safety was acceptable, without evidence of severe hepatotoxicity (92).

In summary, antisense and siRNA approaches all achieve potent suppression of ANGPTL3 with downstream improvements in atherogenic lipoproteins—particularly TG, non–HDL-C, apoB, and LDL-C in patients with dyslipidemia. However, the platforms differ in pharmacology and safety: vupanorsen improved lipid parameters but was limited by dose-related hepatic fat accumulation; zodasiran produced durable lipid reductions without evidence of hepatic fat increase in MRI studies; and solbinsiran achieved apoB lowering of 23–30% and was associated with a reduction in hepatic fat fraction. To date, no randomized trial with MACE endpoints has been completed for these RNA therapeutics. Larger and longer-term studies are required to establish comparative efficacy, long-term safety, and cardiovascular benefits. To facilitate comparison across platforms, **Table 1** summarizes the design, study populations, interventions, lipid outcomes, and cardiovascular endpoints of the key ANGPTL3-targeted therapies discussed above.

**Table 1:** Summary of key clinical and preclinical studies on ANGPTL3 inhibition.

Agent	Trial name	Year	Study type	Experimental subjects	Intervention	Main lipid effects	Cardiovascular outcomes
Evinacumab	Study of Evinacumab (REGN1500) in Caucasian and in Japanese Healthy Volunteers (NCT03146416)	2013–2017	Phase I, randomized, double-blind, placebo-controlled	Healthy Japanese and Western adults	IV 5/15 mg·kg^-^¹ Q4W ×2 or SC 300 mg QW ×8	TG ↓30–70%; LDL-C ↓18–34%; non–HDL-C ↓20–35%; apoB ↓18–33%; HDL-C ↓10–14%	No MACE evaluation; PK/PD and safety only
Evinacumab	Single Ascending Dose Study in Healthy Subjects and Patients With Hypertriglyceridemia (NCT01749878)	2013–2017	Phase I, single ascending dose	Hypertriglyceridemia	Single IV/SC administration	TG ↓47.5%; LDL-C ↓22%; non–HDL-C ↓25%; apoB ↓22%; apoC-III ↓67%	No MACE evaluation
Evinacumab	Multiple Ascending Dose Study in Healthy Subjects and Patients With Hypertriglyceridemia (NCT02107872)	2013–2017	Phase I, multiple ascending dose	Hypertriglyceridemia	SC 300 mg QW; IV 15 mg·kg^-^¹ Q4W	TG ↓65–70%; LDL-C ↓28%; non–HDL-C ↓35%; apoB ↓34%; apoC-III ↓75%	No MACE evaluation
Evinacumab	ELIPSE HoFH: Evinacumab Lipid Studies in Patients With Homozygous Familial Hypercholesterolemia (NCT03399786)	2020	Phase III, randomized, double-blind	HoFH	IV 15 mg·kg^-^¹ Q4W + SOC	LDL-C ↓47%; non–HDL-C and apoB markedly reduced	No MACE endpoint; established LDLR-independent efficacy
Evinacumab	Study of Evinacumab in Patients With Refractory Hypercholesterolemia (NCT03175367)	2020	Phase II, randomized, double-blind	Refractory hypercholesterolemia	IV administration	Maximal LDL-C ↓>50%	No MACE evaluation
Evinacumab	Long-Term Safety and Efficacy in HoFH (NCT03409744)	2023–2025	Open-label extension	Adult and pediatric HoFH	Ongoing IV therapy	Sustained ≈50% LDL-C reduction	Exploratory evidence of reduced CV risk; not RCT endpoints
Evinacumab	Efficacy and Safety of Evinacumab in Pediatric Patients With HoFH (NCT04233918)	2023–2024	Multicenter, open-label	Pediatric HoFH	IV 15 mg·kg^-^¹	Week 24 LDL-C ↓≈48%; non–HDL-C, TC, apoB reduced	No MACE evaluation
Vupanorsen	ISIS 703802 (AKCEA-ANGPTL3-LRx) in Participants With T2DM, NAFLD, and Hypertriglyceridemia (NCT03371355)	2020	Phase IIa, randomized, double-blind	T2DM + NAFLD, moderate HTG	SC 20 mg QW or 40/80 mg Q4W, 24 wks	TG ↓36–53%; ANGPTL3 ↓41–59%; remnant-C ↓38%; non–HDL-C ↓18%; apoB ↓9%	No MACE evaluation
Vupanorsen	TRANSLATE–TIMI 70 (NCT04516291)	2022	Phase IIb, randomized, double-blind	High-risk ASCVD on statins	60–160 mg Q2W or 80–160 mg Q4W, 24 wks	TG ↓41–57%; non–HDL-C ↓22–28%; LDL-C ↓8–16%; apoB ↓6–15%; remnant-C ↓42–59%; hepatic fat ↑ dose-dependently	No MACE; hepatic safety concerns halted program
Zodasiran	AROANG1001: First-in-Human Single and Multiple Dose Study (NCT03747224)	2021–2023	Phase I, basket design	Healthy adults; hepatic steatosis	SC, single or repeat dosing	Day 113: ANGPTL3 ↓64–93%; TG ↓62–72%; non–HDL-C ↓41–49%; LDL-C ↓34–45%; apoB ↓28–39%	No MACE evaluation
Zodasiran	ARCHES-2: Study in Adults With Mixed Dyslipidemia (NCT04832971)	2024	Phase IIb, randomized, double-blind	Mixed hyperlipidemia	SC 50/100/200 mg at Weeks 0 & 12	Week 24: ANGPTL3 ↓54–74%; TG ↓51–63%; non–HDL-C ↓29–36%; LDL-C ↓14–20%; apoB ↓15–22%	No MACE evaluation
Solbinsiran	PROLONG-ANG3: Phase 2 randomized placebo-controlled trial (NCT05256654)	2025	Phase II, double-blind, randomized, placebo-controlled	Adults with mixed dyslipidemia	SC 100/200/400/800 mg on Day 1 ± Day 90 (single or repeat dose)	ANGPTL3 ↓ up to 86%; TG ↓ up to 73%; LDL-C ↓ up to 30%; non-HDL-C ↓ up to 41%; apoB ↓ up to 30%; effects sustained to Day 169	No MACE evaluation; safety acceptable with mostly mild adverse events
CRISPR/Cas9 gene editing	Preclinical studies	2019–2021	–	Mouse models	Adenoviral or LNP-Cas9 editing Angptl3	TG ↓29–37%; LDL-C ↓≈57%; TC ↓≈28%; effect ≥100 days	Reduced atherosclerotic plaque burden
ANGPTL3 Vaccines	Preclinical peptide/VLP vaccine studies	2018–2022	–	Mouse models (ob/ob, Apoe mutant)	Peptide vaccine; Qβ VLP vaccine	TG ↓26–34%; LDL-C ↓24–33%; sdLDL-C ↓34%; improved postprandial TG clearance	Plaque area ↓≈48%; reduced inflammation

Overview of clinical and preclinical studies targeting ANGPTL3, with agents and official trial names (including NCT identifiers) listed separately. Across modalities (monoclonal antibody, ASO, siRNA), ANGPTL3 inhibition consistently lowers triglycerides, non–HDL-C, apoB, and LDL-C—even in LDLR–deficient states. No randomized controlled trial with major adverse cardiovascular events (MACE) as the primary endpoint has yet been completed.

### 5.3 Gene-editing

The clustered regularly interspaced short palindromic repeats (CRISPR)–CRISPR-associated protein 9 (Cas9) platform enables efficient, precise genome editing. Guided by RNA, Cas9 introduces DNA double-strand breaks at target loci, followed by nonhomologous end joining or homology-directed repair. In ANGPTL3 research, editing within exons has yielded durable suppression of protein expression (93, 94). Alexandra et al. employed base editor 3 (BE3) to edit Angptl3 at Gln135 in Neuro-2a cells, using an adenoviral vector (BE3-Angptl3) that achieved a median *in vivo* editing rate of 35%; among ten predicted off-target sites, none showed editing. By day 7, TG had decreased by approximately 37% and TC by 27% compared with controls, with the TG reduction exceeding that observed with BE3-Pcsk9 (95). Adenoviral delivery, however, carries biosafety concerns such as insertional mutagenesis and host immune responses (96). To address these issues, Qiu et al. developed a nonviral lipid nanoparticle (LNP) system enabling hepatocyte-targeted co-delivery of Cas9 mRNA and single-guide RNA, achieving 38.5% Angptl3 editing, a 65.2% reduction in serum ANGPTL3, and decreases in LDL-C and TG of 56.8% and 29.4%, respectively. No editing was detected at nine predicted off-target loci, and no hepatotoxicity or inflammation was observed. Importantly, a single-dose effect persisted for at least 100 days, supporting durable therapeutic potential (97).

### 5.4 Vaccine

Active immunization against ANGPTL3 offers potential advantages—lower cost, better adherence, and longer duration—relative to monoclonal antibodies, ASOs, and siRNAs. Fukami et al. developed a peptide vaccine targeting ANGPTL3 residues 32–41 and demonstrated in ob/ob obese, hyperlipidemic mice that vaccination was associated with reductions in nonfasting TG by approximately 26%, LDL-C by approximately 33%, and small, dense LDL-C by approximately 34%, alongside decreased hepatic lipid deposition, reduced inflammatory markers, and improved nonalcoholic steatohepatitis activity scores. In spontaneously hyperlipidemic Apoe mutant mice, LDL-C and TG decreased by 24% and 32%, respectively, and atherosclerotic plaque area decreased by 48%. After a three-dose series, antibody titers persisted for at least 30 weeks; booster doses at weeks 60 and 90 restored antibody potency and lipid-lowering effects, with detectable titers still present at week 105 and no notable hepatorenal toxicity or histopathology (98, 99).

A virus-like particle (VLP) platform based on Qβ bacteriophage, displaying ANGPTL3 residues 32–47 that encompass the LPL-binding region, elicited high-titer antibodies and reduced steady-state TG by approximately 34% in Balb/c mice. In an oral olive-oil challenge, vaccinated mice exhibited about 60% lower plasma TG over six hours, faster TG clearance, and higher LPL activity; no nonspecific responses to full-length ANGPTL3 were detected, mitigating off-target risk (100).

A liposome-coated mesoporous silica nanoparticle (lipoMSN) system has also been proposed for multiplex CRISPR/Cas9 delivery, including ANGPTL3 as a target. In mice, delivery of a Cas9–guide RNA ribonucleoprotein achieved *in vivo* editing at Angptl3 with a mutation rate of 7.2%. With single-target editing, serum TC decreased by 28.2% and TG by 25%. Combined editing of ANGPTL3 and PCSK9 produced larger effects, with TC decreasing by up to 56.5%, and suggested complementary interactions among ANGPTL3, PCSK9, and APOC3. Delivery efficiency was maintained without liver dysfunction, weight change, or major organ toxicity (101).

Collectively, peptide- and VLP-based vaccines and CRISPR-based nano-delivery systems have demonstrated significant lipid-lowering effects with acceptable safety in animal studies. Translation to humans will require systematic evaluation of immunogenicity, durability, and long-term safety.

## 6 Integrated discussion and clinical translational prospects

Based on the preceding sections, the biological and clinical significance of ANGPTL3 can be summarized into a relatively closed loop. First, ANGPTL3 is primarily liver-derived and secreted, tightly regulated by LXR, insulin, leptin, and post-translational modifications (102). Second, within the “vascular lumen–liver” framework, the ANGPTL3/8 complex and ANGPTL4 dynamically regulate LPL, and by adjusting inhibition of EL, they reshape the cascade from TRLs to remnants to LDL and the function of HDL (52). Third, human LoF variants exhibit a dose–threshold relationship for combined hypolipidemia and reduced coronary risk, indicating a causal effect on lowering apoB exposure and remnant cholesterol (9). Fourth, pharmacologic inhibition reproducibly lowers TG, non-HDL-C, and apoB across platforms and reduces LDL-C even with impaired LDLR function, establishing the clinical feasibility of an LDLR-independent pathway (10). Thus, ANGPTL3 combines mechanistic plausibility with verifiable human evidence and has the potential to move from a candidate lipid-lowering target to a central target for residual risk.

The clearest current application for ANGPTL3 targeting is in HoFH with defective LDLR function. Evinacumab significantly reduces LDL-C and non-HDL-C on top of maximally tolerated standard therapy and is approved for HoFH (10). Next are patients with mixed hyperlipidemia and high TG with high remnant exposure, in whom residual event risk arises more from TRLs and remnant cholesterol, a pathway less affected by conventional LDLR-directed drugs; ANGPTL3 inhibition offers upstream “debulking” and accelerated remnant clearance tailored to this profile (103). For difficult-to-treat patients who remain high in apoB or non-HDL-C despite statins, ezetimibe, and PCSK9 inhibitors, ANGPTL3 inhibition provides complementary pathway targeting.

Preclinical data provide a coherent mechanistic substrate for these translational prospects: decreased Angptl3 expression protects ApoE−/− mice from atherosclerosis (104); antisense inhibition of Angptl3 delays plaque progression while lowering atherogenic lipoproteins and enhancing HDL-mediated RCT (60, 105); and monoclonal antibody blockade reduces TG and non–HDL lipids in mice and primates with endothelial lipase–linked HDL remodeling (106). Together, these findings support an upstream, LDLR-independent route by which ANGPTL3 inhibition reduces TRL burden, accelerates remnant disposal, and improves cholesterol handling, aligning with the clinical lipid phenotype summarized above.

For efficacy assessment and follow-up, a “reduce exposure” strategy should be emphasized, using apoB, non-HDL-C, and remnant cholesterol as primary endpoints, with TG as a pathway marker, and avoiding overreliance on changes in LDL-C or HDL-C alone to infer overall atherogenic exposure (107). When necessary, stable isotope tracers can be incorporated in mechanistic substudies to measure production and fractional catabolic rates of VLDL/IDL/LDL apoB to disentangle contributions of “reduced upstream supply” versus “accelerated remnant clearance.” HDL function should be evaluated by cholesterol efflux capacity, RCT markers, and HDL proteomics rather than inferring dysfunction from lower HDL-C (47). Embedding these dynamic/functional indices into clinical goal-setting can help translate the biological advantages of ANGPTL3 inhibition into actionable treatment decisions.

For population stratification and sequencing of therapies, LDLR function, baseline remnant burden, and overall apoB exposure should be starting points, combined with metabolic status. Patients with impaired LDLR function or those not at goal on maximal conventional therapy should be prioritized for add-on ANGPTL3 inhibition (107, 108). In mixed phenotypes dominated by high TG/high remnants, the initial goal should be upstream debulking and accelerated remnant clearance; evaluate whether ANGPTL3 inhibition produces substantive reductions in non-HDL-C and remnant cholesterol before deciding on parallel or subsequent addition of other pathways. Given complementary mechanisms with other agents, additive reductions in apoB exposure are theoretically achievable. Clinical pathways should predefine thresholds and decision points for add-on intensity (e.g., dual criteria of absolute values and percentage reductions in non-HDL-C and apoB).

Regarding safety monitoring, lower HDL-C under ANGPTL3 inhibition does not equate to impaired HDL function; in high-risk patients on long-term therapy, functional follow-up is advisable for accurate assessment (47). Liver safety should incorporate imaging and biomarkers, distinguishing platform/delivery-specific signals from target effects. Under very low TG or long-term broad hypolipidemia, individualized risk assessment should include nutritional and coagulation indices to avoid indiscriminate discontinuation or overtreatment. In practice, infusion- or injection-related reactions, immunogenicity, drug–drug interactions, and adherence should be recorded to support standardized management. IV monoclonal antibodies suit specialized clinics for high-risk individuals with regular follow-up and intensive management; hepatocyte-targeted nucleic acid drugs, with longer half-lives, may allow extended dosing intervals if approved, improving adherence and enabling collaborative follow-up models. Choice of modality should consider treatment targets, patient preference, affordability, and access to ensure continuity. Institutions are advised to implement unified follow-up templates and electronic reminders centered on apoB/non-HDL-C/remnant cholesterol, with specified blood-draw timing, panels, and thresholds to minimize variability from assay and nutritional status.

Future research must align with the core goal of “event translation.” Currently, randomized controlled trials with MACE as the primary endpoint and adequate follow-up are lacking (107). Subsequent studies should target patients with high apoB/high remnants already on intensive therapy, use non-HDL-C/apoB/remnant cholesterol goal attainment as process endpoints, and adopt MACE as the primary outcome, with kinetic and imaging substudies to calibrate contributions of supply reduction versus clearance acceleration. Standardizing assays for ANGPTL3 and the ANGPTL3/8 complex, harmonizing calculation/reporting of remnant cholesterol, and defining the “minimal effective dose for outcome benefit” will enhance cross-study comparability.

## 7 Conclusion

In summary, ANGPTL3 inhibition combines two key nodes—reducing upstream supply and accelerating remnant clearance—has established clinical value in HoFH with limited LDLR function, and shows clear pathway advantages in high TG/high remnants and refractory high apoB populations. A target framework centered on apoB, non-HDL-C, and remnant cholesterol, together with complementary combination strategies and structured safety monitoring, can advance ANGPTL3 inhibition from “effective lipid lowering” to “precise exposure reduction with predictable translation to outcomes.” If real-world evidence confirms these key elements, ANGPTL3 may become a central target in managing residual cardiovascular risk.
